# Plexin B3 guides axons to cross the midline *in vivo*

**DOI:** 10.3389/fncel.2024.1292969

**Published:** 2024-04-02

**Authors:** Zhi-Zhi Liu, Ling-Yan Liu, Lou-Yin Zhu, Jian Zhu, Jia-Yu Luo, Ye-Fan Wang, Hong A. Xu

**Affiliations:** ^1^Institute of Biomedical Innovation, Nanchang University, Nanchang, China; ^2^School of Basic Medical Sciences, Jiangxi Medical College, Nanchang, China; ^3^The Second Affiliated Hospital of Nanchang University, Nanchang, China; ^4^Jiangxi Provincial Collaborative Innovation Center for Cardiovascular, Digestive and Neuropsychiatric diseases, Nanchang, China

**Keywords:** axon guidance, Semaphorin-Plexin signaling, Plexin B3, Sema3E, Neuropilin1, retinal ganglion cells, autism, neurodevelopmental disorders

## Abstract

During the development of neural circuits, axons are guided by a variety of molecular cues to navigate through the brain and establish precise connections with correct partners at the right time and place. Many axon guidance cues have been identified and they play pleiotropic roles in not only axon guidance but also axon fasciculation, axon pruning, and synaptogenesis as well as cell migration, angiogenesis, and bone formation. In search of receptors for Sema3E in axon guidance, we unexpectedly found that Plexin B3 is highly expressed in retinal ganglion cells of zebrafish embryos when retinal axons are crossing the midline to form the chiasm. Plexin B3 has been characterized to be related to neurodevelopmental disorders. However, the investigation of its pathological mechanisms is hampered by the lack of appropriate animal model. We provide evidence that Plexin B3 is critical for axon guidance *in vivo*. Plexin B3 might function as a receptor for Sema3E while Neuropilin1 could be a co-receptor. The intracellular domain of Plexin B3 is required for Semaphorin signaling transduction. Our data suggest that zebrafish could be an ideal animal model for investigating the role and mechanisms of Sema3E and Plexin B3 *in vivo*.

## Introduction

One of the intriguing questions about the establishment of neural circuits during development is how the axons navigate along appropriate path and find their correct targets. The axons are directed through the brain by a variety of classical guidance cues, including Ephrins, Netrins, Slits, and Semaphorins, and many other non-canonical guidance cues (Dickson, 2002). Semaphorins are a large family of secreted and membrane bound proteins, featured by the presence of a family signature “sema” domain (Raper, 2000). Based on sequence similarities and structural features, Semaphorins are categorized into eight classes. Classes 3 to 7 are mainly found in vertebrates. The class 3 semaphorins (Sema3s), including Sema3A through Sema3G, are secreted proteins, distinct from other Semaphorins that are either transmembranous or membrane-anchored (Raper, 2000). Semaphorins function as either attractive or repulsive cues for migrating cells and growing neurites during neural circuit establishment, depending on the components of the receptor complexes and cellular contexts. Semaphorins are versatile and participate in many biological and pathological processes, such as neural circuit assembly, angiogenesis, cell migration, bone formation, immune response, cancer, and other diseases (Roth et al., 2009; Kang and Kumanogoh, 2013; Rehman and Tamagnone, 2013; Jongbloets and Pasterkamp, 2014; Koropouli and Kolodkin, 2014).

The principal receptors for semaphorins are Plexins, which are grouped into four classes (Plexin A–D). Nine Plexins have been identified in vertebrates, Plexin A1–A4, B1–B3, C1, and D1. Plexins are single-pass transmembrane proteins with an extracellular region interacting with Semaphorins and co-receptors. The intracellular part of Plexins usually contain a GTPase activating protein (GAP) domain and a RhoGTPase binding domain (RBD) that can interact directly with small GTPases (Hota and Buck, 2012; Pascoe et al., 2015). Different Plexin subfamilies can associate and activate or inactivate certain GTPase regulatory proteins (Hota and Buck, 2012). The B family Plexins are unique among Plexins since they possess a C-terminal PDZ-binding motif that can bind directly with leukemia associated RhoGEF (LARG) and PDZ-RhoGEF (Hota and Buck, 2012; Pascoe et al., 2015). Plexin Bs are mainly known for their roles in angiogenesis, heart development, immunity and cancers (Perala et al., 2012), while they are relatively less studied in neural development *in vivo*. It has been shown that Plexin B1 is involved in growth cone collapse *in vitro* (Negishi et al., 2005). Plexin B3 can strongly and B2 can moderately promote neurite outgrowth of primary murine cerebellar neurons (Hartwig et al., 2005). The gene encoding Plexin B3 protein, *PLXNB3*, is associated with verbal performance and white matter development in human brain (Rujescu et al., 2007). Recently, *PLXNB3* mutations were identified in congenital heart disease associated with neurodevelopmental disabilities (Feng et al., 2022), further suggesting that Plexin B3 be important for early neurodevelopment. However, both the morphology and function of the central nervous system seems to be normal in Plexin B3 knockout mice (Worzfeld et al., 2009). Whereas *in vitro* experiments indicate that Plexin B3 functions as a receptor for Sema5A (Artigiani et al., 2004), no similar retinal developmental defects is observed in Plexin B3 mutant mice as that seen in Sema5A mutants (Matsuoka et al., 2011). The lack of neural defects following Plexin B3 mutation in mice suggests alternative models are needed to investigate the role of Plexin B3 in neurodevelopment.

Membranous Semaphorins can bind and signal directly through Plexins, whereas most secreted class 3 Semaphorins require co-receptors such as Neuropilins for signal transduction across plasma membrane (Sharma et al., 2012). Sema3E is an exception among class 3 Semaphorins since it can bind to Plexin D1 directly and Neuropilin is not required for the signaling in certain contexts (Gu et al., 2005; Oh and Gu, 2013). Intriguingly, in some axon navigation, Neuropilin1 exerts a “gating” function in switching the Sema3E-Plexin D1 signaling from repulsion to attraction (Chauvet et al., 2007). The attractant response signaling induced by Sema3E is further shown to be transduced through VEGFR2 (vascular endothelial growth factor receptor type 2) while Plexin D1 functions as a ligand-binding partner together with Neuropilin1 (Bellon et al., 2010). These studies reveal that Sema3E is unique among Sema3s.

We have previously demonstrated that Sema3E is critical for retinal axon guidance at the chiasm in zebrafish (Dell et al., 2013). As aforementioned, the well-established receptor for Sema3E is Plexin D1 (Sharma et al., 2012; Oh and Gu, 2013). However, we detected neither obvious expression of Plexin D1 in retina ganglion cells nor retinal axon guidance defects in Plexin D1 mutants (Dell et al., 2013). Sema3E seems to co-operate with Neuropilin1 in retinal axon guidance (Dell et al., 2013). It is well recognized that the intracellular domain of Neuropilin is too short to transduce signaling. We reasoned that there could be a receptor for Sema3E signaling transduction in retinal axon guidance. In the present study, we started to search for the potential Sema3E receptors. Unexpectedly, we found that Plexin B3 is expressed in retinal ganglion cells and it participates in retinal axon guidance *in vivo*. We also provided evidence suggesting that Plexin B3 together with Neuropilin could function as receptors for Sema3E. Zebrafish could serve as a model organism for investigating the role and mechanism of Plexin B3 and Sema3E *in vivo*.

## Results

### Plexin B3 is expressed in retinal ganglion cell layer when retinal axons are crossing the midline

**FIGURE 1:**
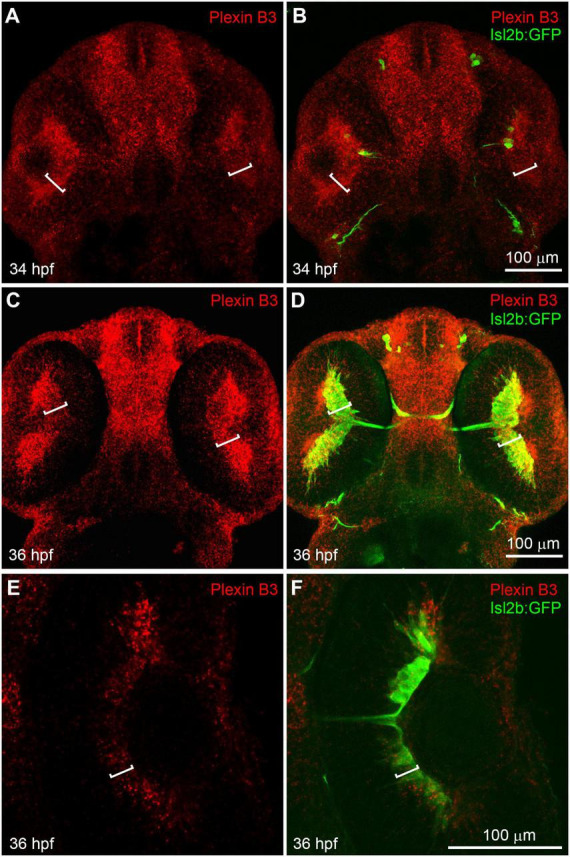
Plexin B3 is expressed in retinal ganglion cells when retinal axons are crossing the midline. *In situ* hybridization was performed on Tg[Isl2b:GFP] transgenic embryos at 34–36 hpf (hour post fertilization), in which the reporter protein GFP is expressed specifically in retinal ganglion cells and can also label retinal axons. **(A)** Fluorescent *in situ* hybridization using complementary RNA probe reveals that Plexin B3 is strongly expressed in retina at 34 hpf. **(B)** Tg[Isl2b:GFP] transgenic labeling demonstrates that Plexin B3 is strongly expressed in retinal ganglion cell layer (square brackets) when pioneer retinal axons are starting to cross the midline and form the chiasm. **(C,D)** Plexin B3 is expressed in retinal axon ganglion cell layer at 36 hpf when many retinal axons cross the midline. **(E,F)** A zoomed in and single plane of confocal image demonstrates that Plexin B3 is expressed in retinal ganglion cell layer. All images are ventral view, rostral to the top. Scale bar, 100 μm.

We have previously found that Sema3E and Neuropilin1a are critical in guiding retinal axons to cross the chiasm in zebrafish (Dell et al., 2013). However, the intracellular domain of Neuropilin is relatively short (about 20 amino acid residues) and it usually functions as a co-receptor by binding Semaphorin ligands in a receptor complex. So, there should be a receptor functioning together with Neuropilin to transduce the Sema3E signal across the cell membrane. The reported mammalian Sema3E receptors in axon guidance include Plexin D1 and VEGFR2 (Bellon et al., 2010; Oh and Gu, 2013). We performed *in situ* hybridization using RNA probes against Plexin D1 and VEGF receptor (VEGFR1, VEGFR2/flk1, VEGFR2/KDRb, and VEGFR3). Neither Plexin D1 nor VEGF receptors seemed to be expressed in neural retina while the VEGF receptors were detected specifically in vessels (Supplementary Figures 1, 2). Moreover, no retinal axon growth defect was detected in Plexin D1 mutants (Supplementary Figure 1; Dell et al., 2013). These results suggested that neither Plexin D1 nor VEGFRs seem to be appropriate candidate receptor for Sema3E. In order to further identify the potential receptor components for Sema3E, we performed *in situ* hybridization with other Plexins (Plexin A1–A4 and B1–B3) (Supplementary Figure 3). Plexin A3 seemed to be expressed at low levels in retinal ganglion cell layers. However, no misprojection of retinal axons was found in Plexin A3 mutants (Dell et al., 2013). Intriguingly, Plexin B3 was found to be highly expressed in retinal ganglion cell (RGC) layer in embryos at 34 h post-fertilization (hpf) (Figures 1A, B). At this embryonic stage, a few newly differentiated retinal ganglion cells are revealed by the transgenic green fluorescent protein (GFP) reporter (Tg[isl2b:GFP]) (Pittman et al., 2008; Poulain et al., 2010) and the pioneer retinal axons are crossing the midline. At 36 hpf, more retinal ganglion cells are labeled by transgenic reporter and many retinal axons cross the midline and form the chiasm. Plexin B3 was mainly expressed in retinal ganglion cell layer at 36 hpf (Figures 1C, D), which was also demonstrated by single plane images (Figures 1E, F). Intriguingly, the spatiotemporal expression pattern of Plexin B3 resembled that of Neuropilin1 (Liu et al., 2004; Dell et al., 2013). Furthermore, Sema3E and Sema3D are expressed in the chiasm at the same time course (Dell et al., 2013). These results indicated that Plexin B3 might be a good candidate for Sema3E receptor component in retinal axon guidance.

### Plexin B3 is required for retinal axons to cross the midline at the chiasm

In order to investigate the role of Plexin B3 in retinal axon guidance, we performed knock down experiments with morpholino antisense oligos. Plexin B3 is highly conserved between zebrafish and mammals. Like human Plexin B3, the zebrafish ortholog also contains an extracellular Sema domain, an intracellular RAS-GAP domain and a PDZ-binding motif (NCBI’s conserved domain database) (Marchler-Bauer et al., 2015). We designed an antisense oligonucleotide morpholino (MO) targeting the fourth exon of zebrafish Plexin B3 (MO^E4^) according to the latest release of zebrafish genome (Ensembl, ENSDART00000026965.11, GRCz11 release 108). The MO was injected into 1–2 cell stage embryos. Sequencing of the cDNA PCR products revealed that the splicing of the fourth exon was interfered (data not shown). The fourth intron was retained in the mature mRNA and premature stop codons were introduced in the reading frame. The deduced protein should be truncated and retain only part of the Sema domain, missing the rest of the protein and lose the function of transducing signals across the plasma membrane.

**FIGURE 2:**
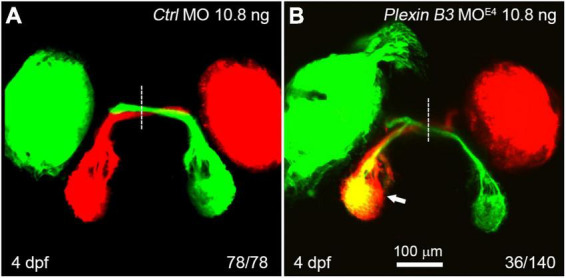
Knocking down Plexin B3 induces retinal axons to misproject into the ipsilateral tectum. **(A)** In control MO treated embryos, all retinal axons cross the midline and project to the contralateral tectum. **(B)** In some of the MO^E4^ treated embryos (36 out of 140 eyes), retinal axons fail to cross the midline at the chiasm and misproject to the ipsilateral tectum (arrow). Images are dorsal view, rostral to the top. Scale bar, 100 μm.

In wild-type zebrafish larvae, almost all of the retinal axons cross the midline and project to the contralateral tectum at 4 days post fertilization (dpf) (Figure 2A). However, in Plexin B3 MO^E4^-treated larvae (morphants), some of the retinal axons failed to cross the midline and projected incorrectly into the ipsilateral tectum (Figure 2B). Although these retinal axons mis-projected into the wrong side of the brain, they still followed the “correct” pathway to join the axons from the other eye (Figure 2B). The ipsilateral misprojections of retinal axons were found in about 26% (36 out of 140) of the examined eyes. We noticed that the ipsilateral misprojections usually occurred in only one eye of an embryo and rarely in two eyes simultaneously, which was also found in our previous studies (Xu et al., 2010; Dell et al., 2013). We have no explanation for this yet. We can only speculate that the antisense morpholino might not be evenly distributed in the embryo. The eye with relatively higher dose of the morpholino will misproject axons, while the eye with lower dose project axons normally. The uneven distribution of morpholino in the embryos might only partially contribute to the asymmetric misprojections since even in the mutants of axon guidance cue receptors, such as robo2 or rb1 mutants, the retinal axons from the two eyes are asymmetrically mis-guided (Fricke et al., 2001; Gyda et al., 2012). These results indicated that Plexin B3 was required for retinal axons to correctly navigate through the chiasm and cross the midline.

### Plexin B3 synergizes with Sema3E and Neuropilin1a in retinal axon guidance

**FIGURE 3:**
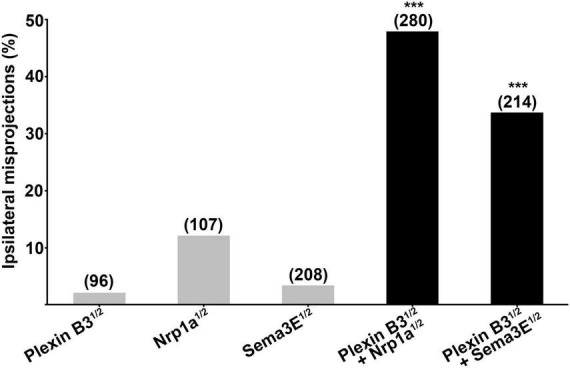
Plexin B3 synergizes genetically with Neuropilin1 and Sema3E in retinal axon guidance. Half doses of Plexin B3, Nrp1a or Sema3E MO results in low proportion of ipsilateral misprojections of retinal axons. Combining half doses of Plexin B3 MO with Nrp1a or Sema3E MO causes dramatic increase of ipsilateral misprojections, which is much more than the simple sum up of the individual half doses of MOs. The combo results strongly indicate that Plexin B3 genetically interact with Nrp1a and Sema3E and might function as receptor-ligand in axon guidance *in vivo*. Stars above the columns indicate that the proportion of eyes with ipsilateral misprojections induced by the combination of half doses is significantly higher than would be expected by assuming that the effects of half doses add together independently. ***p < 0.001, Fisher’s exact test.

The ipsilateral retinal axon guidance errors caused by knocking down Plexin B3 resemble those induced by Sema3E or Neuropilin1a deficiency (Dell et al., 2013), suggesting that Plexin B3 might serve as a receptor for Sema3E in axon guidance. In order to test whether Plexin B3 interacts with Sema3E genetically *in vivo*, we co-injected half doses of morpholinos against both of the genes. The half dose of either Plexin B3 or Sema3E individually induced relatively low percentage of ipsilateral misprojections (less than 5%, Figure 3). However, treating the embryos by combining the half doses of Plexin B3 and Sema3E MO resulted in dramatic increase of ipsilateral misprojections (up to more than 30%), much more than the sum of that caused by the two individual half doses (Figure 3). These results revealed that Plexin B3 and Sema3E function in concert in retinal axon guidance, suggesting that Plexin B3 might serve as a receptor for Sema3E. Similarly, Plexin B3 also synergized with Neuropilin1a (Nrp1a) in guiding retinal axons to cross the midline (Figure 3), suggesting that Nrp1a might function as a co-receptor for Sema3E.

### The cytoplasmic domain of Plexin B3 is required for retinal axon guidance

**FIGURE 4:**
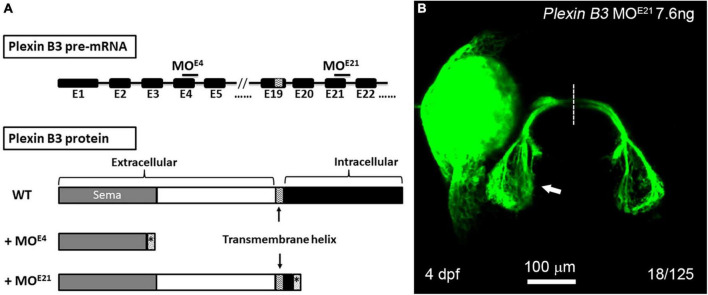
The intracellular domain of Plexin B3 is required for signaling transduction in retinal axon guidance. **(A)** Two morpholinos were designed to interfere with the splicing of exon 4 (E4) and exon 21 (E21) of Plexin B3 pre-mRNA. Sequencing the mis-spliced mRNA revealed that MO^E4^ results in a truncated protein remaining only part of extracellular part and MO^E21^ results in a truncated protein missing the majority of intracellular domains. **(B)** Plexin B3 MO^E21^ treatment causing misprojection of retinal axons into the ipsilateral tectum (arrow), similar as the effect of MO^E4^. The image is dorsal view, rostral to the top. Scale bar, 100 μm.

It is found in mice that the signal of Sema3E is transduced through Plexin D1 or VEGFR2 in neural development (Chauvet et al., 2007; Bellon et al., 2010). However, neither Plexin D1 nor VEGFRs are detected in RGCs of zebrafish embryos, excluding the possibility that they function as Sema3E receptors. Plexin B3 is expressed in RGCs and involved in retinal axon guidance in concert with Sema3E and Nrp1. Considering that the intracellular domain of Nrp1 is too short to transduce signaling, we reason that Plexin B3 might transduce the Sema3E signaling across plasma membrane. In order to test the idea, we designed a second morpholino targeting the exon-intron junction of the 21st exon of Plexin B3, which is immediately downstream of the predicted transmembrane domain of Plexin B3 (Figure 4A). PCR and sequencing results revealed that Plexin B3-MO^E21^ treatment interfered the correct splicing of pre-mRNA and part of the intron was retained in the mis-spliced mRNA (data not shown). The mis-spliced mRNA is predicted to introduce premature stop codons and produce a truncated protein retaining the extracellular and transmembrane helix of Plexin B3 while missing most of the intracellular domains, which are required for signal transduction. The truncated Plexin B3 caused by MO^E21^ is predicted to be retained in the cell membrane since the deduced sequence of the presumed transmembrane helix is highly conserved between zebrafish and higher vertebrates. Knocking down Plexin B3 with the MO^E21^ induced ipsilateral misprojections of retinal axons, similar to those caused by MO^E4^ knockdown (Figure 4B). However, the proportion of ipsilateral misprojection caused by MO^E21^ (18 out of 125 eyes, ∼14.4%) was lower than that caused by MO^E4^ (Figure 2, 36 out of 140 eyes, ∼25.7%), although the proportion was still much higher than control MO (rarely seen ipsilateral misprojection). The lower efficiency of MO^E21^ could be interpreted that the intracellular domain of Plexin B3 might not be so important for signaling transduction. It could also be interpreted as the variable efficiency between morpholinos targeting different parts of the same gene, a similar phenomenon found in our previous studies (Xu et al., 2010; Dell et al., 2013). Overall, the above results suggest that the intracellular domain of Plexin B3 could be important for Sema3E signaling transduction in axon guidance. The treatment with Plexin B3 MO^E4^ and MO^E21^ phenocopied each other also suggested the specific effects of the morpholinos targeting Plexin B3. These results confirmed that Plexin B3 is necessary for retinal axon guidance across the midline and its intracellular domain is required for Semaphorin signal transduction *in vivo*.

### Plexin B3 can bind to Sema3E *in vitro*

**FIGURE 5:**
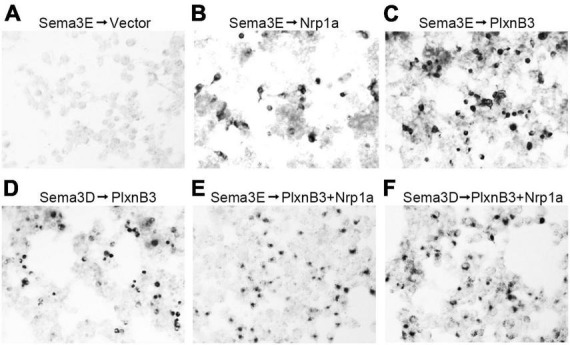
Sema3E binds to Plexin B3 *in vitro*. The binding assay was performed using type 3 Semaphorins tagged with alkaline phosphatase (AP-Sema3) according to He et al. (2019). **(A)** Supernatant containing AP-tagged Sema3E was applied to HEK293 cells transfected with pAG3 empty vector. No obvious binding of Sema3E to the cells was detected. **(B)** AP-tagged Sema3E binds to cells expressing Nrp1a. **(C,D)** Sema3E or Sema3D binds to Plexin B3. **(E,F)** Sema3E or Sema3D binds to the cells co-transfected with Plexin B3 and Nrp1a.

The above results have demonstrated that Plexin B3 could interact with Sema3E and Nrp1a genetically *in vivo*. However, evidence is still required to show the direct signaling from Sema3E to Plexin B3. In order to address the direct interaction between the two molecules, we performed *in vitro* binding assays according to (He et al., 2019). Sema3E could bind to the cells expressing Nrp1a but not those transfected with empty vector, which is consistent with that found by He et al. (2019; Figures 5A, B). Either Sema3E or Sema3D bound to the cells expressing Plexin B3 (Figures 5C, D), suggesting that Sema3E could bind to Plexin B3 directly. We further demonstrated that Sema3E or Sema3D could bind to the cells co-transfected with Plexin B3 and Nrp1a (Figures 5E, F), suggesting that they might also bind to the Plexin B3 and Nrp1a receptor complex. The biochemical binding of Sema3E to Plexin B3 *in vitro* is consistent with the genetic interaction between the ligand and receptor signaling *in vivo*, suggesting that Plexin B3 might function as the receptor to mediate the signal transduction of Sema3E in axon guidance.

## Discussion

In the present study, we demonstrate that Plexin B3 might function as a receptor for axon guidance cue Sema3E in zebrafish. Several lines of evidence support the idea. Firstly, Plexin B3 and its potential ligand and receptor components are spatiotemporally expressed in zebrafish visual system. Among Plexins and VEGF receptors, Plexin B3 is strongly and specifically expressed in retinal ganglion cells when retinal axons are exiting the eyes and crossing the midline (Figure 1 and Supplementary Figures 2, 3). Meanwhile, Sema3E is expressed at the chiasm along the pathways through which the retinal axons navigate (Dell et al., 2013). Furthermore, the potential co-receptor for class three Semaphorins, Nrp1a (He et al., 2019) is also expressed in retinal ganglion cells, similar to Plexin B3 (Liu et al., 2004; Dell et al., 2013). Secondly, no retinal axon projection defects were found in the mutants of Plexin D1 (Supplementary Figure 1), which is a receptor for Sema3E in mice, or that of Plexin A3 (Dell et al., 2013), which seemed to be expressed in retina (Supplementary Figure 3). Thirdly, Plexin B3 deficiency results in ipsilateral misprojections of retinal axons (Figure 2), phenocopying the axon guidance errors caused by impairing Sema3E or Nrp1a (Dell et al., 2013). Fourthly, Plexin B3 genetically interacts with Sema3E and Neuropilin in retinal axon guidance (Figure 3). Fifthly, it has been reported Sema3E could bind directly to Nrp1a *in vitro* (He et al., 2019). We demonstrated here that Sema3E could bind to Plexin B3 as well as the co-transfected receptors Plexin B3 and Nrp1a, suggesting signaling transduction from Sema3E through Plexin B3 (Figure 5). Finally, the intracellular domain of Plexin B3 is required for signal transduction in retinal axon to cross the midline (Figure 4). All the above data are consistent with the idea that Plexin B3 functions as a receptor for Sema3E in zebrafish axon guidance.

Our finding that Sema3E signaling through Plexin B3 but not Plexin D1 in retinal axon guidance in zebrafish seems to be different from the reported Sema3E/PlexinD1 signaling in axon guidance in mice. Similarly, it has been demonstrated that Sema3E genetically interacts with Plexin B2 instead of Plexin D1 in zebrafish angiogenesis and the Sema3E/Plexin B2 signaling antagonizes Plexin D1 in endothelial cells (Lamont et al., 2009). The different receptors employed by Sema3E in axon guidance and angiogenesis could be attributed to the difference between their expression, function or species.

The ipsilateral projections of retinal axons found in binocular animals are caused by repulsive molecules expressed at the chiasm, such as Ephrin B2 (Nakagawa et al., 2000). However, the contralateral routing of axons has long been presumed to be a default program since the axons grow straight forward. Several molecules can promote the growth of retinal axons, such as CD44, L1, SSEA-1, and particularly two secreted proteins Vax1 and Sfrp1/2 (Secreted Frizzled-Related Proteins) expressed in chiasm cells (Prieur and Rebsam, 2017). However, these molecules seem to promote retinal axon growth but not guidance decisions. Recent evidence has demonstrated that midline crossing of retinal axons is an active process instead of a passive one (Prieur and Rebsam, 2017). The retinal axons need to overcome the inhibitory environment of the chiasm to cross the midline. Only a few signaling pathways have been found to promote retinal axons to cross the midline, including VEGF/Nrp1 (Erskine et al., 2011; Tillo et al., 2015) and Sema6D/Nr-CAM/PlexinA1 (Kuwajima et al., 2012) in mice. However, the disruption of these signaling pathways only causes a small portion of the retinal axons to misproject ipsilaterally, indicating that other redundant signals exist to promote and ensure midline crossing. It has been found that Sema3Fa and Sema3Gb are expressed dorsal to the optic chiasm (Callander et al., 2007) while Sema3H is expressed in the region of the optic nerve head in zebrafish (Stevens and Halloran, 2005). Functional studies are required in the future to address whether these guidance cues are involved in midline crossing at the chiasm. The Sema3E/Nrp1/Plexin B3 signaling in zebrafish found in this study seems to be permissive or attractant for retinal axons to cross the midline. It is not known yet whether the signaling pathway exists or functions to promote chiasm formation in higher vertebrates. It has been revealed that the cells expressing class 3 Semaphorins, including Sema3E, are located far from the chiasm in mice (Erskine et al., 2011; Kuwajima et al., 2012). It has been found that the VEGF signaling but not Sema3 signaling through Nrp1 is essential for retinal axons to cross the midline (Erskine et al., 2011). However, no evidence directly excludes Sema3E from participating in retinal axon development yet. Sema3E has been found to be expressed in a subpopulation of mouse retinal ganglion cells (Fukushima et al., 2011; Kim et al., 2011). Moreover, Sema3E can collapse the growth cones of chick and rat retinal axons *in vitro* (Steinbach et al., 2002; Steffensky et al., 2006; Sharma et al., 2014). Further studies are definitely required to examine whether Sema3E is involved in retinal axon guidance in higher vertebrates and to identify its potential receptors.

The reports on the roles of Plexin B3 in neural development are still controversial. It is demonstrated that Plexin B3 is expressed selectively in white matter and oligodendrocytes (Worzfeld et al., 2004). However, the morphology of the central nervous system and behavior of the Plexin B3 mutant mice are indistinguishable from the wild type controls (Worzfeld et al., 2009). It is speculated that the normal phenotype of the Plexin B3 mutant mice could be due to redundant Semaphorin/Plexin signaling pathways functioning in the nervous system. It has been demonstrated that Plexin B3 is expressed not only in glia (Worzfeld et al., 2004) but also in neurons (Cheng et al., 2001; Hartwig et al., 2005). Plexin B3 is found to promote axon growth *in vitro*, probably through homophilic interaction (Hartwig et al., 2005). Our current findings suggest that Plexin B3 functions as a receptor for Sema3E, although the possibility of homophilic Plexin B3 interaction can’t be excluded. The only reported ligand for Plexin B3 so far is Sema5A (Artigiani et al., 2004), however, it still remains to be examined whether the Plexin B3-Sema5A signaling functions in neural development. Previous evidence demonstrates that Sema5A might function as a ligand for Plexin A3 in zebrafish axon guidance (Hilario et al., 2009). Further study is required to clarify whether Sema5A is expressed in zebrafish visual system and whether it serves as a ligand for Plexin B3 in axon guidance. It has been found that human Plexin B3 mutations are associated with white matter volume and cognitive performance (Rujescu et al., 2007). It would be interesting to investigate whether the pairing of Plexin B3 with Sema3E or other potential ligands in axon development is conserved between lower vertebrates and humans.

The signaling of class 3 semaphorins is usually transduced through Plexin A or Plexin D1 receptors with Neuropilin functioning as a co-receptor. Sema3E is an exception among Sema3s since it has been shown to bind Plexin D1 directly in the absence of Neuropilin. The participation of Neuropilin in Plexin B signaling has not been reported before. Our results suggest that Nrp1 might function as a co-receptor facilitating the binding of Sema3E to Plexin B3 and modulating the signaling transduction through Plexin B3. There are several lines of evidence to support the speculation. Firstly, the intracellular domain of Neuropilin rarely transduces signaling. Secondly, the intracellular domain of Plexin B3 is required for signaling transduction (Figure 4). Thirdly, Sema3E has been demonstrated to bind Nrp1 *in vitro* (Figure 5B). Fourthly, Nrp1 can modulate the sensitivity of retinal axons to Sema3s at the midline and consequently facilitate axon crossing in the chiasm (Dell et al., 2013). Finally, the expression level of Nrp1 is regulated by cAMP (Dell et al., 2013), which is a second messenger downstream of G-protein coupled receptors that can modulate the sensitivity of retinal axons to midline guidance cues (Xu et al., 2010). Further studies are required to examine the role of Nrp1 in facilitating the signaling transduction of Sema3E-Plexin B3 in axon guidance.

Our results reveal that the cytoplasmic part of Plexin B3 is required for signal transduction in axon guidance. Upon binding of Semaphorins to Plexins, the cytoplasmic parts of Plexins dimerize and lead to activation of downstream signaling cascades. The intracellular domains of Plexin B3 are highly conserved between zebrafish and higher vertebrates. The intracellular region of B type Plexins is composed of a juxtamembrane segment, a RBD and a GAP) domain, which are shared by other Plexins. Besides these common domains, B Plexins also contain a unique C-terminal PDZ-binding motif “VTDL,” which binds to guanine nucleotide exchange factors (GEFs), PDZ–RhoGEF, and LARG (Pascoe et al., 2015). These small GTPases and GEFs are critical mediators for Plexins in axon growth and guidance through regulating cytoskeleton, cell adhesion and endocytosis. Besides these small molecules, Plexins can also interact with other molecules such as collapsin-response mediator proteins (CRMPs). CRMP4 has been found to interact with Sema3E tripartite receptor complex and its cytoskeleton-binding domain is required for Sema3E’s axon growth-promoting activity (Boulan et al., 2021). We have demonstrated that CRMP4 is critical for zebrafish retinal axon growth and guidance (Liu et al., 2018). It is tempting to speculate that CRMP4 could be involved in the Sema3E-Plexin B3 signaling in zebrafish retinal axon guidance. The involvement of CRMP4 and Plexin B3 mutations in neuropsychiatric disorders (Rujescu et al., 2007; Tsutiya et al., 2017) could make zebrafish a valuable model organism to further investigate their roles and the underlying mechanisms in neurodevelopment and neuropsychiatric disorders.

## Materials and methods

### Zebrafish husbandry

Wild-type AB strain zebrafish were raised and maintained at 28.5°C on a 14/10 h light/dark cycle. Heterozygous transgenic Isl2b:GFP fish (Pittman et al., 2008) were kindly provided by the Chi-Bin Chien Lab, University of Utah. Adult fish under 12 months of age were mated to produce embryos. Embryonic and larval fish were raised in E3 embryo medium by adding 0.006% phenylthiourea at 22–24 hpf to prevent pigmentation. All zebrafish study was performed following the protocols approved by the Nanchang University Animal Care and Use Committee.

### *In situ* hybridization

Fluorescent *in situ* hybridization was performed as described previously (Xu et al., 2010) with minor modifications. Briefly, antisense complementary RNA probes were digoxigenin-labeled and incubated with embryos to detect the expression pattern of various transcripts. The horse-radish peroxidase (POD)-conjugated Anti-DIG antibody (Roche; catalog #11 207 733 910) was applied to detect the DIG-labeled cRNA probes and the signal was amplified with a cyanine 3-coupled tyramide system (PerkinElmer; NEL 744). Immunostaining was performed simultaneously where needed to detect the co-localization of GFP-labeled retinal neurons and axons.

### Retinal axon labeling

Zebrafish larvae were collected at 4 or 5 dpf and fixed in 4% paraformaldehyde. Lipophilic dyes, DiD, and DiI (D7757 and D282 from Thermo Fisher), were separately micro-injected into the retinal ganglion cell layer of the two eyes. The dyes were allowed to diffuse along retinal axons overnight at 4°C or 1–2 h at 37°C. The fluorescently labeled retinal projections were acquired on a confocal laser scanning microscope (Olympus FV1000). The images were presented as maximum projection of z-stacks.

### Morpholino design and microinjection

Morpholino (MO) antisense oligonucleic acids were ordered from Gene-Tools, LLC (Philomath, OR, USA). Two MO oligos were designed to block the splicing of Plexin B3 (Transcript ID ENSDART00000026965.11, GRCz11, Ensembl release 108) at the exon-intron junctions of exon 4–intron 4 (Plexin B3 MO^E4^) and exon 21–intron 21 (Plexin B3 MO^E21^). The sequence of the two MOs are: Plexin B3 MO^E4^, 5′-AGA CAG GTG AGC TCC AGT ACC TGC C-3′; Plexin B3 MO^E21^, 5′-AAC AAA GGC ACT GAA CTG ACC CGT A-3′. Other MOs used in this study were ordered according to published sequences: Nrp1a, 5′-GAA TCC TGG AGT TCG GAG TGC GGA A-3′ (Lee et al., 2002; Dell et al., 2013); Sema3E, 5′-TTG TAG AGA TGA ACA CTT ACG GTA G-3′ (Dell et al., 2013). Usually 1–2 nl of the MOs at desired concentrations were microinjected into the yolk of 1–2 cell stage zygotes.

### AP-tagged protein binding assay

The ligand-receptor binding assay was performed with alkaline phosphatase (AP)-tagged protein as described (Kobayashi et al., 1997; He et al., 2019). Briefly, HEK293 cells were inoculated in the DMEM with high glucose (Gibco, NY, USA) and incubated at 37°C with the supplement of 5% CO_2_. After the cells grew to almost 80% confluence, 1 μg of plasmid encoding AP-Sema3 was added and transfected using Lipofectamine 3000 transfection kit (Thermo Fisher Scientific, MA, USA) for 4–6 h. The cells were changed into fresh medium and let recovery overnight. The temperature was lowered to about 30°C and the supernatants of AP-Sema3 transfected HEK293 cells were harvested 24–48 h later and stored at −80°C.

The Plexin B3 or Nrp1a transfected HEK293 cells were used for Sema3 binding assay. The supernatant of pAG-Plexin B3 and/or pAG-Nrp1a transfected HEK293 cells was removed and the cells were washed with PBS for three times. Then, the transfected cells were incubated with the above collected supernatants for 1 h at room temperature. Then, the supernatants were removed and the cells were fixed with Acetone/Formaldehyde fixative (60% acetone, 3% formaldehyde, 20 mM HEPES, pH 7.0) for 10 min. After washing with PBS three times, the cells were heated at 65°C for 90 min. The cells were then incubated with BCIP/NBT substrate (Sangon, Shanghai, China) in Alkaline Phosphatase buffer [100 mM Tris–HCl (pH 9.5), 100 mM NaCl, 5 mM MgCl_2_] at 37°C in the dark. The stained cells were visualized under a Nikon AZ100 microscope. Images were captured using a Nikon DIGITAL SIGHT DS-Fil1 digital camera and processed with NIS-Elements F 3.0 (Nikon).

## Data availability statement

The original contributions presented in this study are included in this article/Supplementary material, further inquiries can be directed to the corresponding author.

## Ethics statement

The animal study was approved by the Nanchang University Animal Care and Use Committee. The study was conducted in accordance with the local legislation and institutional requirements.

## Author contributions

Z-ZL: Funding acquisition, Investigation, Methodology, Supervision, Validation, Writing – review and editing. L-YL: Investigation, Validation, Visualization, Writing – review and editing. L-YZ: Investigation, Validation, Writing – review and editing. JZ: Investigation, Validation, Writing – review and editing. J-YL: Methodology, Validation, Visualization, Writing – review and editing. Y-FW: Investigation, Methodology, Validation, Writing – review and editing. HX: Conceptualization, Data curation, Funding, acquisition, Methodology, Project administration, Supervision, Validation, Visualization, Writing – original draft, Writing – review and editing.

## Supplementary material

Supplementary Figure 1 Plexin D1 is dispensable for retinal axon guidance at the chiasm. Whole mount fluorescent *in situ* hybridization was performed with cRNA probe against Plexin D1 on Tg[Isl2b:GFP] transgenic embryos in which GFP reporter protein is specifically expressed in retinal ganglion cells and retinal axons. **(A,B)** Plexin D1 seems to be only weakly, if there is any, expressed in retinal ganglion cells at 36 hpf when retinal axons are crossing the chiasm. **(C,D)** The homozygous mutation of Plexin D1 does not affect the midline crossing of retinal axons at the chiasm.

Supplementary Figure 2 Vascular endothelial growth factor (VEGF) receptors are expressed restrictedly in the vessels of the eyes. Whole mount fluorescent *in situ* hybridization was performed with cRNA probes against VEGF receptors (VEGFR1, VEGFR2a/Flk1, VEGFR2b/Kdr, and VEGFR3) on Tg[Isl2b:GFP] transgenic embryos. The VEGF receptors are expressed in the vessels of the eyes and the chiasm.

Supplementary Figure 3 The expression of Plexin As, Plexin B1, and Plexin B2 are rarely detected in retinal ganglion cells. Whole mount fluorescent *in situ* hybridization was performed with complementary RNA probes against Plexin As (Plexin A1a, Plexin A1b, Plexin A2, Plexin A3, and Plexin A4), Plexin B1 and Plexin B2 on Tg[Isl2b:GFP] transgenic embryos. The expression of these Plexins can be detected in the eyes but are rarely detected in RGC layers except Plexin A3.
