# Supplementary figures and images for: Plexin B3 guides axons to cross the midline in vivo
Source: Front Cell Neurosci. 2024 Apr 2;18:1292969. doi: 10.3389/fncel.2024.1292969 (PMC11018898; doi:10.3389/fncel.2024.1292969)

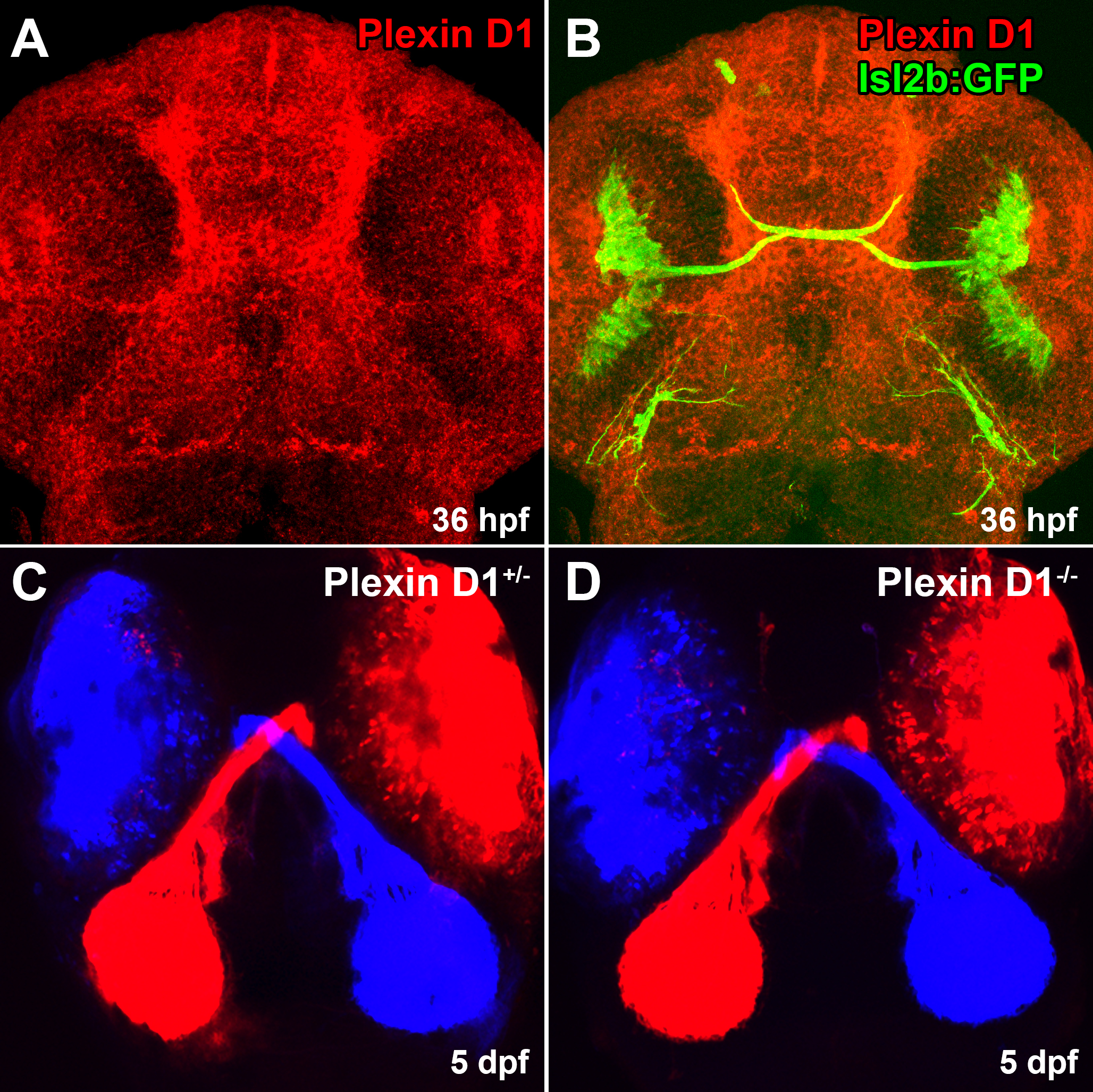

Supplement: Supplementary Figure 1 — Plexin D1 is dispensable for retinal axon guidance at the chiasm. Whole mount fluorescent in situ hybridization was performed with cRNA probe against Plexin D1 on Tg[Isl2b:GFP] transgenic embryos in which GFP reporter protein is specifically expressed in retinal ganglion cells and retinal axons. (A,B) Plexin D1 seems to be only weakly, if there is any, expressed in retinal ganglion cells at 36 hpf when retinal axons are crossing the chiasm. (C,D) The homozygous mutation of Plexin D1 does not affect the midline crossing of retinal axons at the chiasm. [file Image_1.JPEG]

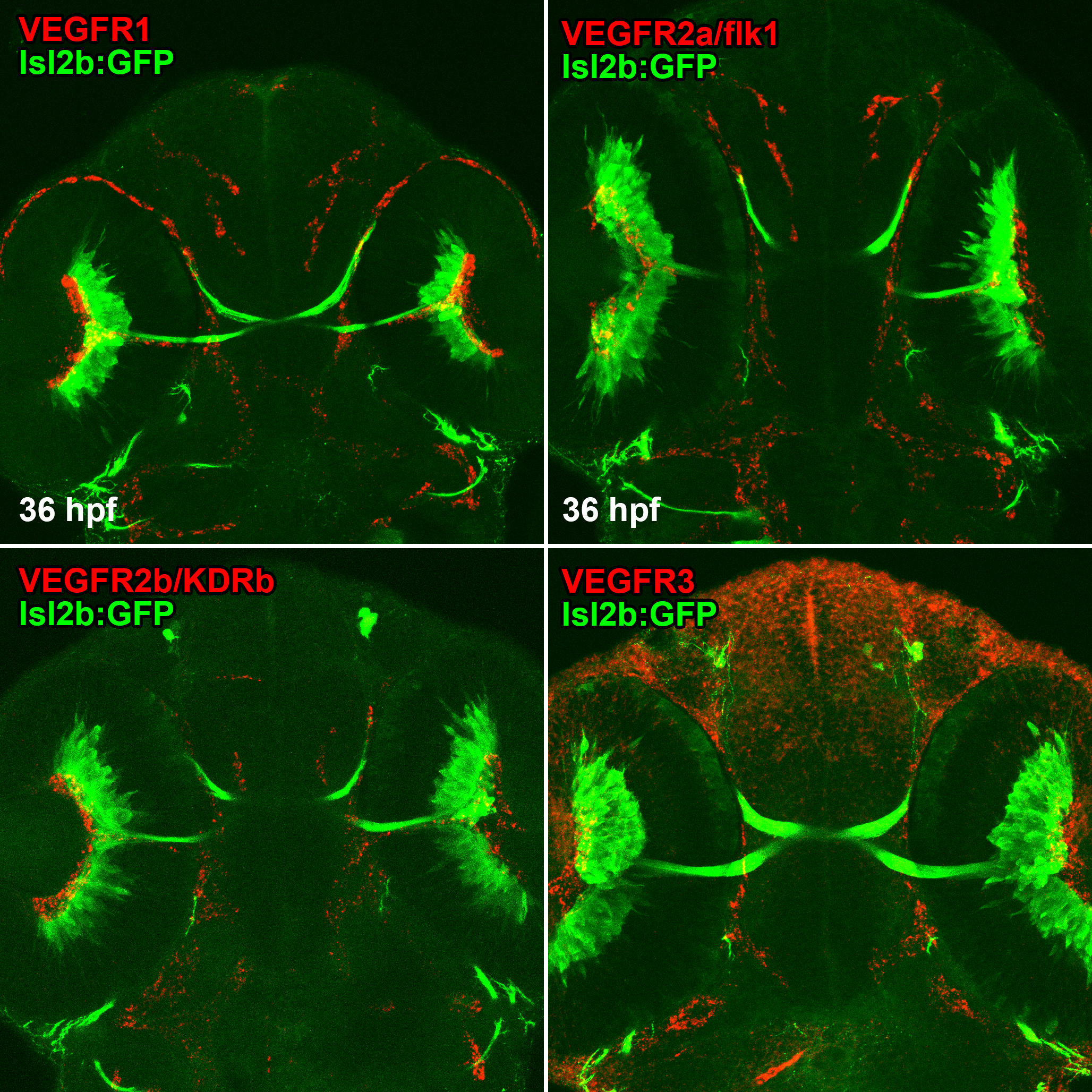

Supplement: Supplementary Figure 2 — Vascular endothelial growth factor (VEGF) receptors are expressed restrictedly in the vessels of the eyes. Whole mount fluorescent in situ hybridization was performed with cRNA probes against VEGF receptors (VEGFR1, VEGFR2a/Flk1, VEGFR2b/Kdr, and VEGFR3) on Tg[Isl2b:GFP] transgenic embryos. The VEGF receptors are expressed in the vessels of the eyes and the chiasm. [file Image_2.JPEG]

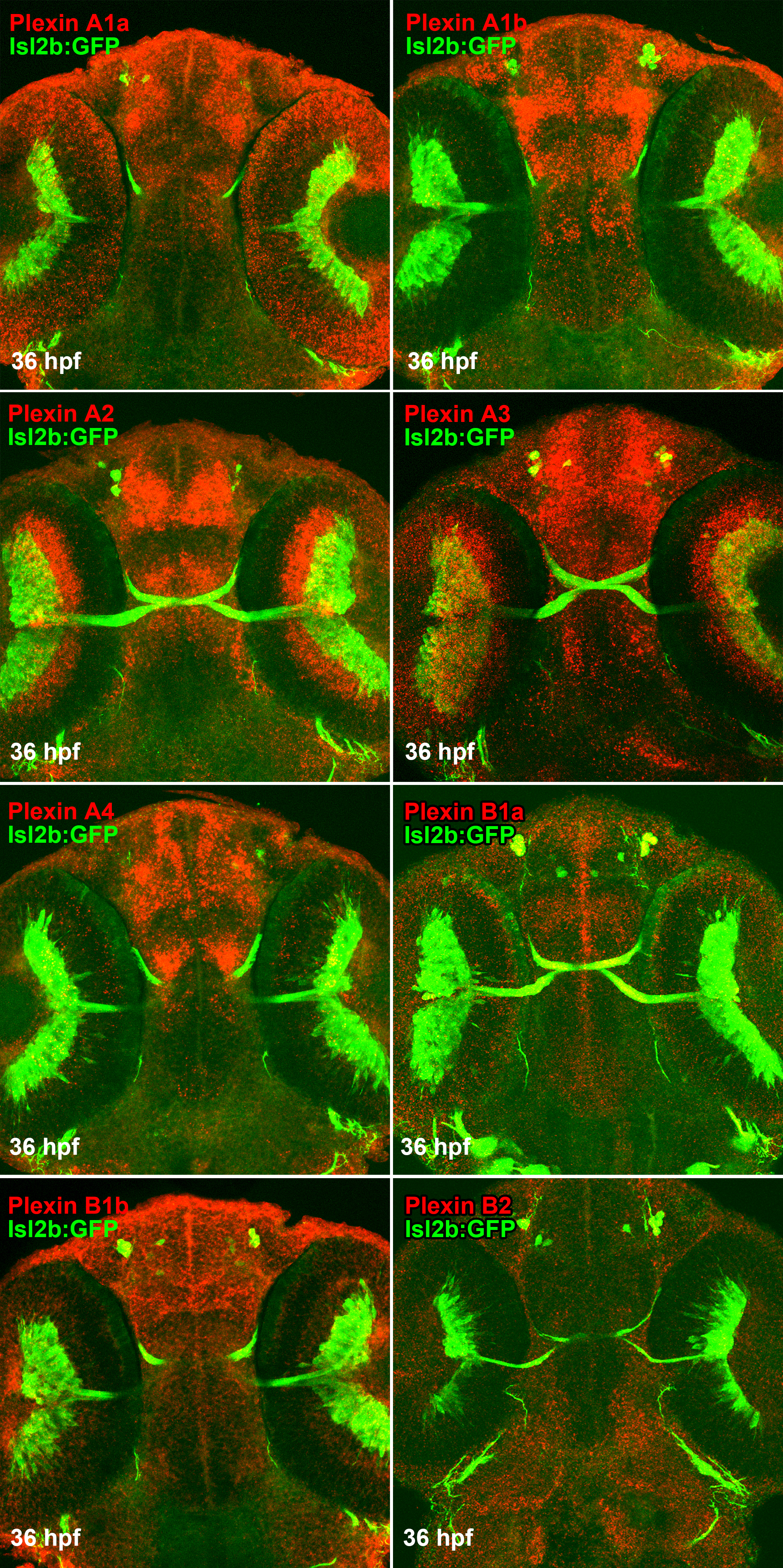

Supplement: Supplementary Figure 3 — The expression of Plexin As, Plexin B1, and Plexin B2 are rarely detected in retinal ganglion cells. Whole mount fluorescent in situ hybridization was performed with complementary RNA probes against Plexin As (Plexin A1a, Plexin A1b, Plexin A2, Plexin A3, and Plexin A4), Plexin B1 and Plexin B2 on Tg[Isl2b:GFP] transgenic embryos. The expression of these Plexins can be detected in the eyes but are rarely detected in RGC layers except Plexin A3. [file Image_3.JPEG]
